# Development and validation of the climate change-related reproductive concerns scale (CCRCS)

**DOI:** 10.1016/j.joclim.2024.100351

**Published:** 2024-10-11

**Authors:** Matteo Innocenti, Gabriele Santarelli, Chiara Comerci, Niccolò Carluccio, Enrico Anzaghi, Chiara Cadeddu

**Affiliations:** a Italian Climate Change Anxiety Association (AIACC), Florence, Italy; b Section of Hygiene, University Department of Life Sciences and Public Health, Università Cattolica del Sacro Cuore, Rome, Italy; c Erasmus School of Health Policy and Management, Erasmus University Rotterdam, the Netherlands

**Keywords:** Eco-anxiety, Eco-paralysis, Climate change anxiety, Climate change worry, Climate change-related reproductive concerns scale, Climate change-related reproductive concerns

## Abstract

**Introduction:**

As global concerns about climate change intensify, emerging research reveals a link between climate change anxiety and individuals' decisions regarding parenthood. More people are choosing not to have children due to worries about their carbon footprint or the future implications of climate change on their offspring. This trend emphasizes the critical necessity for a nuanced comprehension of how environmental concerns intertwine with reproductive intentions. To address this imperative, our study develops the Climate Change-related Reproductive Concerns Scale (CCRCS) and evaluates its psychometric properties.

**Methods:**

CCRCS was developed and validated in a sample of 206 Italian adults aged 19 to 51. Ten items were created to evaluate climate change-related reproductive attitudes: 5 anti-reproductive items and 5 pro-reproductive, with their responses reversed for consistency in interpretation.

**Results:**

Exploratory factor analysis revealed a single-factor structure, explaining 63.82 % of the variance, with the scale demonstrating good internal consistency (α = 0.85). The factor structure was replicated, and the scale's validity was examined through correlations with measures of eco-paralysis and climate change anxiety, with significant correlations supporting construct validity. Furthermore, the relationship between climate change-related reproductive concerns and adaptation responses was explored, assessing the impact of framing on CCRCS scores.

**Conclusion:**

The CCRCS provides a reliable and valid measure of these concerns, highlighting the psychological impact of climate change anxiety on reproductive decision-making and emphasizing the need for nuanced understanding in this area.

## 1 Introduction

In recent years, there has been an unprecedented global focus on climate change, with scientists highlighting human activities such as pollution, overpopulation, urbanization, and biodiversity loss as major contributors to the crisis [1,2,3].

While traditionally seen as an issue primarily affecting the environment, it is now increasingly clear that climate change significantly impacts human well-being. The rising temperatures, the proliferation of water-borne diseases, the increase in malnutrition, and the surge in Extreme Weather Events (EWEs) like wildfires, floods, and heat waves are among the most visible consequences [4,5]. These effects intertwine with insidious environmental changes such as prolonged droughts, reduced livability, and amplified sociopolitical challenges in certain regions, leading to increased disease transmission, food insecurity, migration, inter-group conflicts, and economic inequities [4,5].

While the physical impacts of climate change are well-documented, its psychological effects have received less attention. The experience of climate change and EWEs can lead to psychological disorders such as post-traumatic stress disorder (PTSD), depression, and anxiety, as well as other issues such as sleep disorders and cognitive impairments [6,7]. These findings suggest that as climate change effects become more apparent globally, particularly with the increasing frequency of EWEs, more people are likely to experience these symptoms [8].

Climate change does not just impact the mental health of those directly affected, it also affects individuals concerned about its significant threat. The emotional toll of such a devastating global issue often includes feelings of sadness, fear, anger, powerlessness, helplessness, guilt, shame, despair, hurt, grief, and depression, particularly among younger people [9,10,11]. Additionally, a considerable portion of youth globally express experiencing functional difficulties and holding pessimistic views about the future. These beliefs include concerns about humanity's failure to protect the planet, a frightening outlook for the future, a sense of doom for humanity, reduced access to opportunities compared to previous generations, the destruction of valued aspects of life, feeling threatened in terms of security, and hesitancy towards starting families [10].

Young people are increasingly experiencing anxiety related to climate change, a phenomenon named “climate change anxiety” [9], characterized by emotion-driven rumination behaviors, obsessive thinking, and disruptions to sleep and appetite [12,13]. In contrast, “climate change worry” refers to a more specific and persistent concern about imminent and future threats posed by climate change, including uncontrollable changes in the ecosystem and potentially disastrous effects, often leading to constant anticipation of these threats [14,15].

Climate change anxiety, as well as climate change worry, can act as powerful drivers for individuals to combat the climate crisis, stimulating them to engage in pro-environmental behaviors (PEBs), defined as voluntary actions taken by individuals or groups intended to benefit or minimize harm to the environment [16]. On the other hand, the worry and anxiety may lead to feelings of helplessness, hopelessness, and a loss of motivation, ultimately eroding individuals' confidence in their ability to enact change [17,18,19], and even to eco-paralysis, a phenomenon marked by apathy and passivity, where individuals feel overwhelmed and unable to take action due to their environmental concerns [20,21,22]. Eco-paralysis could also discourage individuals from participating in PEBs, instilling a sense of hopelessness about the future [19], where feelings of being overwhelmed and an inability to act hinder proactive engagement. This sense of helplessness and hopelessness may consequently push individuals towards extreme measures [18], like opting for fewer or no children, as they grapple with reconciling personal values with environmental concerns. This highlights the profound impact of climate change anxiety on reproductive attitudes, emphasizing the need for comprehensive strategies addressing psychological well-being and environmental sustainability.

Recent literature shows that the anxiety surrounding the future is significantly influencing reproductive attitudes, indicating a link between increased anxiety levels and a decreased desire to become parents [23,24]. Moreover, media discourse on the question "Is it okay to have a child?" reflects a growing sense of fear and anxiety about bringing children into a world facing environmental degradation [25].

Factors contributing to this decline in desire for children include concerns about a child's carbon footprint, overpopulation, or uncertainty about the future [26,27,28,29]. A poll conducted in the USA among 20 to 45-year-olds [30] revealed that 33 % of respondents who either had or expected to have fewer children than they desired cited "worries about climate change" as one of the motivating factors. Namely, 39 % of the sample interviewed in a large study involving 10,000 children and young people in ten countries [10] stated that they were hesitant to have children.

Scholars are now emphasizing the significant influence of population growth on individuals' decisions regarding parenthood, drawing attention to the interplay between fertility, population dynamics, and climate change. While historical concerns about not having children focused on fears about population growth, recent research in this area is limited [29]. Scientific evidence indicates a close correlation between population size and greenhouse gas emissions, with population growth being a key driver of emissions increase, and suggesting that slower population growth could result in a substantial reduction in total emissions by 2100 [31]. As a result, environmentally conscious individuals are increasingly taking this connection into account when making reproductive decisions [29]: “I cannot produce another person that will continue to destroy the planet, as they will inherit my first-world lifestyle. I also cannot live with the feeling of responsibility that I made a decision to have a child for my own pleasure while destroying exactly what I'm fighting to save” (childfree 32 years old [29]).

Choosing not to have children is increasingly seen as a way to reduce the carbon footprint of the traditional Western family model [25]. Although interestingly, Schneider-Mayerson and Leong [29] found that in the 'Global North', concerns about climate impacts on children outweigh worries about their carbon footprint in reproductive decisions. Moreover, younger respondents were particularly concerned about potential climate-related impacts on their offspring compared to older respondents. This apprehension about the future is a recurring theme in the literature on young people's perceptions of the climate crisis and parenthood decisions. For example, an Australian study [32] revealed that a significant portion of 10–14-year-olds believed the world might end during their lifetime due to climate change and other global threats. Consequently, reproductive choices may be influenced by concerns about exposing children to climate change consequences or by a belief in imminent catastrophe: “It wouldn't be fair to have a child knowing what I know about the climate crisis, I would feel guilty” (young patient [13]).

In 2018, the United Kingdom saw the emergence of the "BirthStrike" movement, initiated by activist Blythe Pepino. This campaign was a call to action, urging individuals to pledge not to have children as a means of protesting the insufficient measures taken to combat climate change. The movement highlighted a critical ethical issue: the responsibility of citizens in post-industrialized nations to consider the environmental impact of their reproductive choices. However, "BirthStrike" faced significant criticism for potentially echoing narratives of population control that have historically been tainted with racism and classism [25]. Discussions around overpopulation intersect with various sensitive topics like reproductive rights, politics, and culture. Some argue that overpopulation is exaggerated and unfairly blames marginalized groups for environmental issues [25,26]. Moreover, social science research increasingly frames the decision to have children as an individual choice, similar to other consumer decisions such as reducing meat consumption or using a car.

The emergence of the BirthStrike movement highlights the complexities of discussing reproduction within the context of the Anthropocene. This movement brings to the fore the delicate issue of choosing not to have children due to concerns about climate change, and it raises critical questions about how to approach this topic without perpetuating gendered violence or objectifying women's bodies. Media narratives that focus on reproduction often adopt a precarious stance, suggesting that individuals bear the responsibility for mitigating climate change. This approach is particularly problematic in the Global South, where it is imbued with moral overtones. Such rhetoric risks diverting attention from more systemic issues, such as the vastly disproportionate consumption patterns of the world's wealthiest 1 % compared to those in the Global South [25]. While larger families may be more common in the latter, their overall consumption remains significantly lower. It is essential to shift the discourse from individual blame to a broader analysis of systemic factors, including wealth inequality and consumption habits. These systemic issues have a far greater environmental impact than the carbon footprint associated with having children. Addressing these root causes is vital for creating effective and equitable solutions to the environmental challenges we face [25].

Concerns about climate impacts on children stem from expectations, hopes, and fears about the future, which are future-oriented due to the delay between greenhouse gas emissions and climatic changes. Reproduction is closely tied to envisioning the future, influencing reproductive intentions and choices over time [29,32]. With growing awareness of climate change's implications on reproductive attitudes, individuals face complex ethical dilemmas when considering parenthood in a changing world. This decision, once deeply personal, is now intertwined with concerns about environmental sustainability and future generations' well-being, especially for women in post-industrialized countries [33].

In Italy, the demographic landscape is characterized by a significant decline, with birth rates plummeting to an alarming low of 1.18 children per fertile woman [34]. This trend is the culmination of multifaceted factors: economic hurdles, steep living expenses, rigid work conditions, evolving cultural values, entrenched gender disparities, and the looming threat of climate change's disruptive effects. Thus, it is imperative to examine how concerns about climate change are influencing reproductive decisions among Italians. To mitigate these downward demographic trends, there is a pressing need for comprehensive strategies that go beyond the scope of the modest economic incentives currently offered under stringent conditions.

Given these considerations, our study aims to develop and validate a tool in Italian for assessing the relationship between climate change anxiety and reproductive decision-making, the Climate Change-Related Reproductive Concerns Scale (CCRCS). We aim to explore the interplay between reproductive attitudes and climate change-related psychological constructs, including climate change worry, climate change anxiety, and eco-paralysis, to elucidate their correlations and potential influences. Furthermore, this study investigates the potential mediating role of climate change-induced functional and cognitive impairments on reproductive attitudes. Through these objectives, the study seeks to contribute to a deeper understanding of the complex dynamics between climate change perceptions and reproductive decision-making.

Understanding these dynamics can inform policymakers, healthcare professionals, and individuals for more informed family planning decisions, contributing to sustainable population policies. This validation study is a crucial step towards comprehensively understanding this interplay and empowering individuals to make informed choices in the face of a changing climate, leading to more resilient and sustainable societies grounded in the stewardship of the planet for future generations.

## 2 Methods

### 2.1 Participants

Following Nunnally's recommendations of 10 respondents per item, we determined that our a priori targeted sample size would be at least 100 participants. The original sample was composed of 253 subjects; however, we excluded 47 subjects who did not complete the survey, resulting in a final sample of 206 participants from 19 to 51 years of age (*M* = 25,93, *SD* = 4,79). The vast majority was composed of people assigned “female” at birth (*n* = 173, 83 %), while the rest was “male” assigned (*n* = 33, 17 %). The conditions for participating in this study were: a minimum age of 18 and a maximum age of 80 years, Italian nationality, and having residency in Italy. The socio-demographic characteristics of the sample are shown in Table 1. All participants provided informed consent prior to participating in the study.

**Table 1:** Descriptive statistics for variables in the study.

	Mean	SD
**Age**	24.96	4.78
**Education (years)**	15.31	1.89
**CCRC**	27.06	8.26
**MHC Total**	41.25	9.53
Emotional Well Being	9.42	2.40
Social Well Being	11.65	3.58
Psychological Well Being	20.19	4.77
**CCWS total**	27.97	7.59
**DASS depression**	13.60	4.82
**DASS anxiety**	11.56	4.16
**DASS stress**	16.43	4.57
**Eco-Paralysis**	27.06	7.96
**CCAS Cognitive Impairment**	20.79	8.02
**CCAS Functional Impairment**	11.59	4.53

*N* = 253.

### 2.2 Climate Change-related Reproductive Concerns Scale (CCRCS) Development

Ten items were created to evaluate climate change-related reproductive attitudes: 5 anti-reproductive items (e.g. “I don't want to have children: the main reason is that they'll grow up in a world ruined by climate change”) and 5 pro-reproductive, reversed items (e.g. “I am not afraid to have children because I don't think future generations will be significantly impacted by climate change."). To develop a scale measuring concerns about having children due to climate change, the authors conducted a narrative literature review on fear related to this issue and on the potential impacts of climate change on future generations which revealed that climate change-related reproductive attitudes are often associated with the fear of children facing the consequences of climate change or concerns about a child's carbon footprint [13,25,28,29]. This finding is consistent with research highlighting the significant influence of climate change anxiety on reproductive decision-making [23,24]. Thus, in the development of the instrument concerns about a child's carbon footprint, overpopulation, and uncertainty about the future were considered, ensuring they captured the range of cognitive, behavioral, and affective concerns associated with climate change and reproductive decision-making.

Two researchers (M.I. & G.S.) independently generated 10 statements each, which were thoroughly reviewed anonymously. Afterwards, a third researcher (C.Co.) selected 10 statements from the combined pool considering item clarity, relevance, and comprehensiveness, while ensuring they were non-redundant and suitable for measuring cognitive, behavioral, and affective concerns. The initial researchers approved the final version. Redundant items were removed and a balanced mix of positively and negatively worded statements were retained. This resulted in a 10-item questionnaire along a 5-point Likert scale ranging from 1 = “strongly disagree” to 5 = “strongly agree”, where a higher score indicates stronger anti-reproductive attitudes (i.e., less intention of having children in the future), while a lower score indicates stronger pro-reproductive attitudes (i.e., more intention of having children in the future; see **Appendix A**). The "don't know" option was included to discourage casual responses and provide participants with the opportunity to express uncertainty or lack of knowledge.

### 2.3 Supplementary Instruments

#### 2.3.1 Climate Change Anxiety Scale (CCAS)

The Climate Change Anxiety Scale (CCAS; Italian version: [34]) developed by Clayton and Karazsia [35] was used to assess climate change anxiety. The CCAS is a self-reported questionnaire with 13 items that measure the perception of anxiety in relation to climate change. It is divided into two subscales: items 1–8 compose the “cognitive impairment scale”, while items 9–13 compose the “functional impairment scale”. This first factor refers to having trouble remembering or learning new things, making decisions, or other factors that affect daily life (“thinking about climate change makes it difficult for me to sleep”). The second factor refers to the limitations a person can have, indicating behavioral engagement (“my concerns about climate change interfere with my ability to get work or school assignments done”). Participants needed to evaluate the frequency with which they experienced the situation described by the item along a Likert scale from 1 to 5 (1 = Never; 2 = Rarely; 3 = Sometimes; 4 = Often; 5 = Almost always). Regarding reliability, the scale showed good internal consistency, both for the Cognitive Impairment subscale (α = 0.78) and the Functional Impairment subscale (α = 0.73).

#### 2.3.2 Climate Change Worry Scale (CCWS)

To evaluate climate change worry, we used the Italian version of the Climate Change Worry Scale (CCWS; [37]) originally developed by Stewart [38] to evaluate proximal worry related to climate change, focusing on individual apprehensions rather than broader social or global consequences. The CCWS is a self-report measure that focuses on the frequency of worry related to possible proximal manifestations of climate change, such as extreme weather events and effects on people close to the respondent, rather than examining broader issues such as inter-group conflicts or resource scarcity. The questionnaire is composed of 10 items, each one concerning one aspect of proximal and personal worry about climate change (“I worry about how climate change may affect the people I care about”). Respondents need to evaluate how frequently each statement applies to themselves along a 5-point Likert scale (1 = Never; 2 = Rarely; 3 = Sometimes; 4 = Often; 5 = Always). The scale items showed high internal consistency (Stewart, 2021), as well as good test-retest reliability (*r* = 0.91).

#### 2.3.3 Depression Anxiety Stress Scale (DASS-21)

The Depression Anxiety and Stress Scale (DASS 21; [39]; Italian version: [40]) is a 21-item scale developed to discriminate between symptoms of depression, anxiety, and stress. It has three scales, each one composed of seven items: the scale “Depression” (items 3, 5, 10, 13, 15, 16, 21) assesses low self-esteem, lack of incentive, and dysphoria; “Anxiety” (items: 2, 4, 7, 9, 14, 18, 19) assesses somatic and subjective symptoms of anxiety and acute fear; and “Stress” (items 1, 6, 8, 11, 12, 13, 17) measures tension, irritability, difficulty in relaxing, and agitation. Moreover, a general distress score can be calculated by summing the values of all three subscales. Respondents need to evaluate how often in the last week the sentences applied to them were: 0 = “Did not apply to me at all”, 1 = “Applied to me to some degree, or some of the time”, 2 = “Applied to me a considerable degree, or a good part of the time”, and 3 = Applied to me very much, or most of the time. DASS-21 scores were calculated by summing the values of the 7 items that compose every subscale (range for each subscale: 0 - 21), while the general distress score was calculated by summing the values of all three subscales (range: 0 - 63). The DASS-21 is an efficient and economical questionnaire that allows to assess three dimensions at the same time. Regarding reliability, the DASS-21 showed high internal consistency (α_TOT_ = 0.93).

#### 2.3.4 Mental Health Continuum Short Form (MHC-SF)

The Mental Health Continuum Short Form (MHC-SF; [41]; Italian version: [42]) is a self-assessment questionnaire that measures three aspects of well-being: emotional, psychological, and social. The Mental Health Continuum Long Form (MHC-LF) is composed of 40 items while the MCH-SF has 14 items: 3 for emotional (hedonic) well-being, 6 for psychological, and 5 for social well-being. All these aspects combined compose the eudaimonic well-being, defined as “the subjective experiences associated with eudaimonia or living a life of virtue in pursuit of human excellence” [43]. Respondents need to indicate how often they feel the emotion/experience expressed in the statement using a 6-point Likert scale ranging from 0 = “never” to 5 = “every day”. The MHC-SF demonstrated high internal consistency in our sample (α_TOT_ = 0.95; α_EMOT_ = 0.88; α_PSYCH_ = 0.90; α_SOCIAL_ = 0.90).

#### 2.3.5 Eco-Paralysis Scale (EPS)

The Eco-Paralysis Scale (EPS) is a self-assessment questionnaire developed by Innocenti et al. [19] to assess various key features of eco-paralysis outlined by Albrecht [17] such as behavioral inhibition, feelings of hopelessness about the future, and a sense of helplessness.

The instrument delineates individuals' beliefs regarding their role in and positive impact on climate change, as well as their reactions of paralysis when confronted with it. These observations were derived from interviews with individuals who self-reported experiencing eco-paralysis.

The EPS comprises 11 items, with respondents indicating their level of agreement on a 5-point Likert scale ranging from 1 = "strongly disagree" to 5 = "strongly agree". Higher scores suggest a greater tendency toward experiencing eco-paralysis in one's life. The EPS demonstrated high internal consistency in our sample (α = 0.98).

### 2.4 Procedure

Recruitment for the study utilized convenience and snowball sampling methods. Initially, 45 participants were recruited by distributing the research protocol through the Italian Climate Change Anxiety Association (AIACC) newsletter. To address potential selection bias inherent in non-probabilistic sampling, each participant was then requested to share the questionnaire with five additional individuals until data saturation was reached. Participants who completed the survey were later contacted for a follow-up after three months to minimize recall bias. Data collection took place between January and June 2023, utilizing the Google Forms platform. Participants were first briefed on the study's objectives, procedures, potential risks, and benefits. They were informed about their rights, including the right to withdraw at any time without penalty. Written informed consent was obtained from each participant, ensuring they understood the study's purpose, the voluntary nature of their participation, and the measures in place to protect their confidentiality. Participants were also informed that their data would be anonymized and that any identifying information would be removed. Participants were made aware that the study would be published and accessible on an open-access basis. Consent was also obtained for the publication of their data and any potential use of their photos or other images. The study received ethics approval from the Comitato Etico di Area Vasta Centro (CEAVC) of Tuscany (Area Vasta Centro - Protocol n. 20042/OSS of 13/06/21).

### 2.5 Validity Assessment

Regarding the development and validation of the Climate Change-related Reproduction Attitude Scale, the following hypotheses were assessed:

1. Hypothesis 1. We hypothesized a positive correlation between climate change-related reproductive concerns and climate change anxiety, both functional and cognitive impairment.
2. Hypothesis 2: In order to assess convergent validity, we also hypothesized a positive correlation between climate change-related reproductive concerns and climate change worry, given that this emotion reflects a sense of resignation in the face of environmental degradation.
3. Hypothesis 3: Climate change anxiety was shown to be correlated to clinical measures of depression and anxiety [35,36], therefore another positive correlation was hypothesized between climate change-related reproductive concerns and a clinical measure of anxiety, depression, and stress, as well as with emotional, social and psychological wellbeing.
  Hypothesis 4: Previous research [19] showed a positive correlation between climate change anxiety and eco-paralysis in an Italian adult sample, therefore we hypothesized a positive correlation between eco-paralysis and attitudes towards reproductive decisions related to climate change. Eco-paralysis indicates the inability to engage in forward-thinking actions, suggesting that this could be applied in starting a family. Therefore, we hypothesized the presence of a positive and indirect effect of climate change anxiety (both cognitive and functional impairment) on reproductive concerns, with eco-paralysis serving as a mediator for this effect.

### 2.6 Data Analysis

Internal consistency was assessed by estimating Cronbach's alpha coefficient for the scales considered. Hypotheses made to assess validity were tested through partial correlations adjusted for sex and age. The factorial structure of the Climate Change-related Reproductive Concerns Scale (CCRCS) data was tested through confirmatory factor analysis. Exploratory factor analysis was used to assess the factorial structure of the CCRCS. In this analysis, an Eigenvalue is calculated for each factor which is extracted. The Eigenvalue represents the variance associated with each factor [44]. The scree test and parallel analysis were used to select the number of factors. The scree test consists of a graph that represents the decreasing curve of eigenvalues and allows the selection of factors that precede the flattening of the curve [45]. This method has shown good reliability in identifying the strongest eigenvalues despite the subjectivity of the method [46]. On the other hand, we did not select factors with an eigenvalue greater than 1 because this method has been shown to select too many factors [47].

A mediation model was used to test the hypothesis that the effect of cognitive and functional impairment caused by climate change anxiety on climate change reproductive attitudes are mediated by Eco Paralysis. The model was age- and instruction-adjusted.

Statistical analyses were performed using IBM SPSS 25.0, AMOS 24, and R version 4.2.0 (22 April 2022), with p values < 0.05 indicating statistical significance.

## 3 Results

### 3.1 Descriptive Statistics

The descriptive statistics for the variables in the study, including the mean and standard deviation, are presented in Table 1.

### 3.2 Psychometric properties of the Climate Change-related Reproductive Concerns Scale (CCRCS)

The descriptive statistics for each item of the Climate Change-related Reproductive Concerns Scale (CCRCS), including the mean, standard deviation, and item-total correlation, are presented in Table 2.

**Table 2:** Mean, standard deviation, and item-total correlation for each item in the Climate Change-Related Reproductive Concerns Scale (CCRCS).

Item	Mean	SD	Item-total correlation
**1**	2.90	1.02	0.76
**2**	2.70	1.01	0.75
**3**	2.70	1.06	0.71
**4**	2.60	1.08	0.74
**5**	2.67	1.03	0.73
**6**	2.78	0.99	0.79
**7**	2.70	1.05	0.72
**8**	2,66	0.99	0.74
**9**	2.65	1.00	0.73
**10**	2.68	1.06	0.79

*N* = 253.

An Exploratory Factor Analysis (EFA) was carried out to assess the factorial structure of the Climate Change-related Reproductive Concerns Scale (CCRCS). The analysis revealed that only one factor had an Eigenvalue greater than 1, as shown in Table 1. Additionally, the scree plot (Fig. 3) indicated an elbow point after one factor. Both the Eigenvalue criterion and the scree plot suggested that a single-factor model adequately fits the data.

The single-factor structure explains 63.82 % of the total variance. All items correlate positively with the single factor. Table 4 presents the factor loadings for each item of the CCRCS. Internal consistency was evaluated for the single-factor scale (Cronbach's alpha = 0.937)

**Fig. 1:**
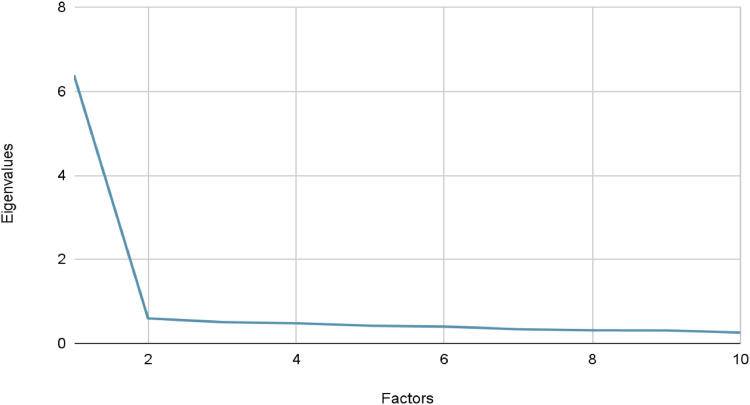
Scree test used for the EFA of the Climate Change-Related Reproductive Concerns Scale (CCRCS). *Note. N=253*.

CCRCS showed a positive correlation with the eco-paralysis scale (*r* = 0.971, *p* < 0.001), the Cognitive Impairment Subscale of CCAS (*r* = 0.856, *p* < 0.001), the Functional Impairment Subscale of CCAS (*r* = 0.737, *p* < 0.001), and the DASS Depression Subscale (*r* = 0.129, *p* = 0.04); no correlation was detected with either the MHC subscales or the total score (MHC Emotional Wellbeing *r* = −0.012, *p* = 0.845, MHC Social Wellbeing *r* = −0.088, *p* = 0.162, MHC Psychological Wellbeing *r* = −0.004, *p* = 0.955, MHC total score *r* = −0.039, *p* = 0.535), Climate Change Worry Scale (*r* = 0.024, *p* = 0.725), DASS Anxiety (*r* = 0.117, *p* = 0.062), DASS Stress (*r* = 0.052, *p* = 0.413) (Fig. 1, Table 3).

**Table 3:** Factor and Eigenvalues for the EFA.

Factor	Eigenvalue
**1**	6.38
**2**	0.59
**3**	0.51
**4**	0.48
**5**	0.42
**6**	0.40
**7**	0.34
**8**	0.31
**9**	0.31
**10**	0.26

*N* = 253.

**Table 4:** Item loadings for the Climate Change-Related Reproductive Concerns single-factor model.

Item	Loading
**1**	0.815
**2**	0.801
**3**	0.767
**4**	0.791
**5**	0.786
**6**	0.838
**7**	0.780
**8**	0.791
**9**	0.781
**10**	0.836

*N* = 253.

### 3.3 The interplay between climate change anxiety, eco-paralysis, and climate change-related reproductive concerns

**Fig. 2:**
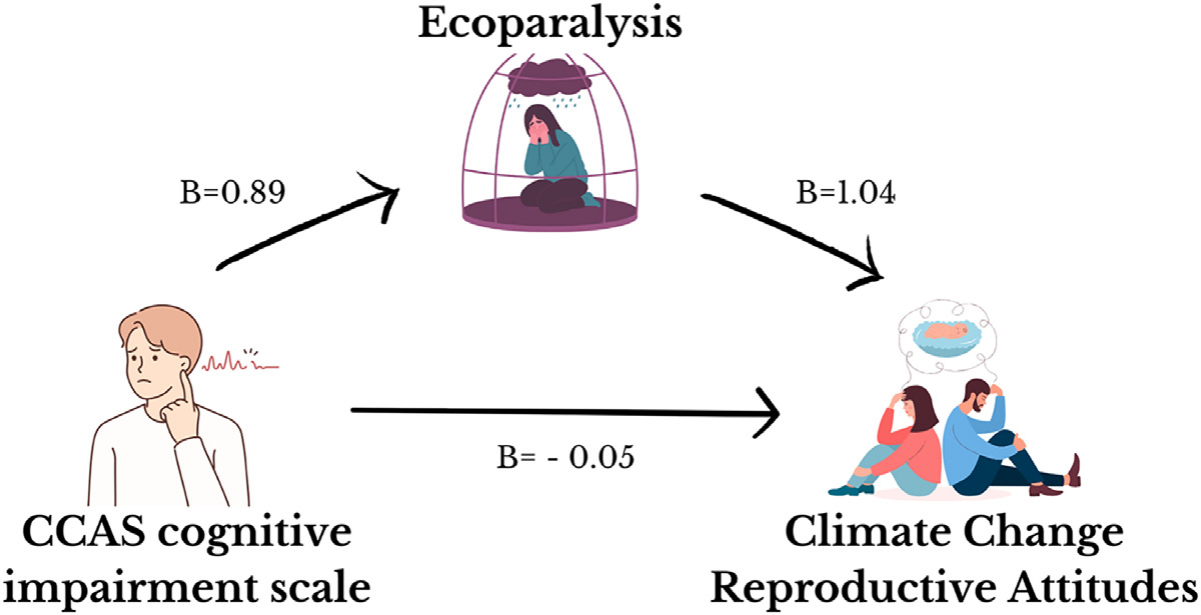
Direct and indirect effect of Climate Change Anxiety's Cognitive Impairment on Climate Change Reproductive Concerns. *Note. B* = regression coefficient.

The result of the sex- and instruction-adjusted ANCOVA using the CCAS cognitive impairment subscale as the independent variable and Eco Paralysis total score as the dependent variable was significant (F(3235) = 303.26, *p* < 0.001, R2 = 0.79), and the effect of CCAS cognitive impairment on Eco Paralysis total score was negative and significant (*B* = 0.89, *t* = 29.69, *p* < 0.001). The results of the sex- and age-adjusted ANCOVA using the CCAS cognitive impairment and Eco Paralysis scale total score as independent variables and CCRCS total score as the dependent variable was significant (F(4234) = 990.17, *p* < 0.001, R2 = 0.94). The effect of cognitive impairment related to climate change anxiety on CCRCS total scores was not significant (*B* = −0.05, *t* = −1.66, *p* = 0.09), and the effect of the Eco Paralysis total score on the CCRCS total score was positive and significant (*B* = 1.04, *t* = 29.95, *p* < 0.001). Therefore, climate change anxiety had no direct effect (direct effect = −0.05) and a significant positive indirect effect (indirect effect = 0.93, Bootstrap Standard Error = 0.04, Bootstrap I.C.: 0.84; 1.02) on CCRCS mediated by Eco Paralysis scores (Fig. 2).

**Fig. 3:**
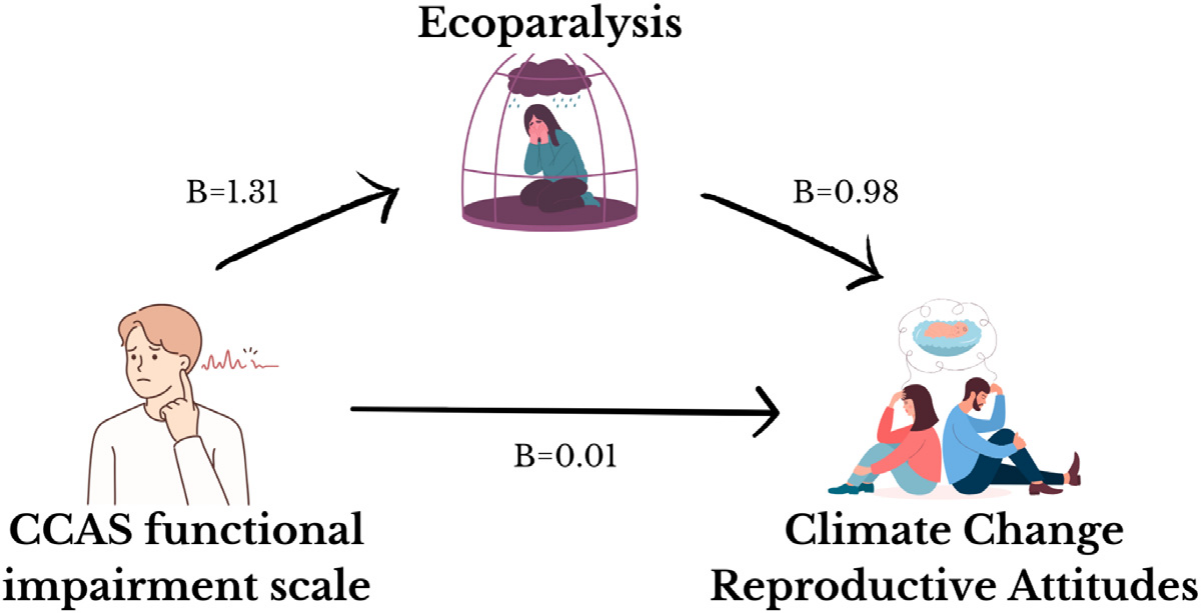
Direct and indirect effect of Climate Change Anxiety's Functional Impairment on Climate Change-related Reproductive Concerns. *Note. B* = regression coefficient.

A mediation model was used to test the hypothesis that the effect of functional impairment caused by climate change anxiety on climate change reproductive attitudes is mediated by Eco Paralysis. The model was age- and instruction-adjusted. The results of sex- and instruction-adjusted ANCOVA using the CCAS functional impairment subscale as the independent variable and Eco Paralysis total score as the dependent variable was significant (F(3235) = 104.07, *p* < 0.001, R2 = 0.57), and the effect of CCAS functional impairment on Eco Paralysis total score was positive and significant (*B* = 1.31, *t* = 17.28, *p* < 0.001). The results of sex- and age-adjusted ANCOVA using the CCAS functional impairment and Eco Paralysis scale total score as independent variables and CCRCS total score as the dependent variable was significant (F(4234) = 978.47, *p* < 0.001, R2 = 0.94). The effect of cognitive impairment related to climate change anxiety on CCRCS total scores was not significant (*B* = 0.01, *t* = 0.37, *p* = 0.70), and the effect of Eco Paralysis total score on CCRCS total scores was positive and significant (*B* = 0.98, *t* = 40.67, *p* < 0.001). Therefore, climate change anxiety had no direct effect (direct effect = 0.01) and a significant positive indirect effect (indirect effect: 1.29, Bootstrap Standard Error = 0.08, Bootstrap I.C.: 1.12; 1.47) on CCRCS mediated by Eco Paralysis scores (Fig. 3).

## 4 Discussion

To better understand attitudes towards climate change and its effect on reproductive decisions, a fundamental step involves the development of appropriate tools. In this study, a 10-item scale was developed, specifically tailored to assess individuals' attitudes to this topic. The questionnaire was congruent with existing literature on climate change perceptions and reproductive health decision-making [13,23,24,26,28,29]. However, to ensure its validity and reliability, rigorous psychometric testing was conducted. In this discussion, we elaborate on the process of questionnaire development, highlight key findings, and address implications for future research and practice.

The present study provides robust support for the psychometric properties of the newly developed Climate Change-related Reproductive Concerns Scale (CCRCS). Reliability analysis revealed good internal consistency, and the scale demonstrated a single-factor structure. The hypotheses made to assess validity were confirmed and underscored the intricate relationship between climate change anxiety, eco-paralysis, and reproductive attitudes, offering insights into individuals' responses to environmental challenges.

Firstly, our correlation analyses showed significant positive associations between the Climate Change-related Reproductive Concern Scale (CCRCS) and measures of climate change anxiety and eco-paralysis. Specifically, CCRCS showed strong positive correlations with the eco-paralysis scale and both subscales of the Climate Change Anxiety Scale (CCAS) cognitive impairment and functional impairment. As shown in recent literature [23,24,26], these findings underscore the pervasive influence of climate change anxiety and its related impairments on individuals' attitudes toward reproductive behavior concerning climate change. Climate change anxiety is increasingly recognized as a significant psychological phenomenon, encompassing a range of cognitive, emotional, and behavioral responses to the perceived threat of climate change [10,35]. Our findings suggest that individuals experiencing higher levels of climate change anxiety in terms of cognitive and functional impairments are more likely to exhibit eco-paralysis—a phenomenon characterized by feelings of helplessness, being overwhelmed, and inaction in the face of environmental challenges [19,48]. This aligns with previous research highlighting the adverse psychological effects of climate change anxiety and its potential to hinder adaptive responses to environmental threats [35], and the crucial role of eco-paralysis in mediating this relationship [19,49].

Moreover, our analyses indicated a significant positive correlation between CCRCS and the DASS Depression Subscale, suggesting a link between depressive symptoms and attitudes toward reproductive behavior related to climate change. Depression, often co-occurring with anxiety disorders, may exacerbate feelings of hopelessness and disengagement from environmental issues, further contributing to eco-paralysis, signifying a broader pessimistic outlook encompassing self, world, and future—aligning with Beck's [50] classical cognitive triad of depression. However, it is important to note that the strength of this association was comparatively weaker [51] than those observed with climate change anxiety and eco-paralysis, indicating that while depressive symptoms may play a role, climate change anxiety appears to be a more salient factor in shaping attitudes towards reproductive behavior concerning climate change. Climate change anxiety emerges as the predominant emotion documented across scientific articles, newspaper reports, interviews, documentaries, and blogs concerning the climate crisis [52]. This anxiety stems from the inherent unpredictability, uncertainty, and uncontrollability associated with climate change [21,52], making it potentially more pertinent to reproductive decisions than depression. The pervasive uncertainty about the future and the inability to control climate impacts pose challenges for individuals, particularly young people, in planning long-term commitments such as starting a family [52]. This feeling of powerlessness and the perceived threat to future prospects contribute to the classification of climate change as an existential threat [21,28,52], thereby reinforcing the connection between anxiety and reproductive decision-making.

Conversely, our analyses did not reveal significant correlations between CCRC and measures of general mental health, climate change worries, and clinical measures of anxiety and stress. This suggests that attitudes towards reproductive behavior related to climate change may be influenced more strongly by specific dimensions of climate change anxiety (such as cognitive and functional impairments) rather than broader mental health factors or general anxiety symptoms. These results prompt further exploration into the nuanced relationship between climate change-related reproductive attitudes and mental health indicators in the context of climate change concerns. Future research may delve into additional factors that could influence reproductive attitudes and consider longitudinal designs to capture potential changes over time.

The mediation analysis conducted, with the data to be interpreted as preliminary, allows us to understand the role of Eco-paralysis in reproductive concerns associated with climate change. Our analysis demonstrated significant relationships: climate change anxiety's cognitive impairment had a negative effect on eco-paralysis scores, while functional impairment had a positive effect. Eco-paralysis, in turn, mediated the relationship between functional impairment and attitudes towards reproductive behavior, showing that higher levels of eco-paralysis led to more pronounced reproductive choices influenced by climate change concerns. Preliminary data from the mediation analyses highlight that the only component of climate change anxiety that can influence reproductive concerns is eco-paralysis, perhaps by hindering proactive engagement in environmental actions and fostering skepticism about the future [19]. Indeed, cognitive impairment may lead to a decreased sense of eco-paralysis, potentially indicating a heightened state of alertness or cognitive engagement with environmental issues [19,53]. In contrast, functional impairment, reflecting practical limitations in individuals' ability to cope with climate change-related stressors may exacerbate feelings of eco-paralysis and hinder adaptive responses, indicating a sense of helplessness or inaction in the face of environmental threats [19,54,55]. Climate change anxiety is described by several authors as an emotion that can both stimulate pro-environmental behaviors or induce paralysis from action through eco-paralysis [19]. Individuals who experience anxiety concerning climate change usually have more awareness and might adopt behaviors to reduce their impact on the world. Consequently, climate change anxiety could be a form of motivation to act against the climate crisis but has also the potential to become a source of eco-paralysis, suppressing people from taking action [21]. Anxiety can be paralyzing, thus eco-anxiety can cause helplessness, loss of hope, depression, loss of motivation, and nullification of the sense of efficacy [19,20,21,22], in turn influencing reproductive behaviors.

### 4.1 Limitations

This study should be interpreted in the context of some limitations. The moderation analysis conducted was part of a pilot study rather than an observational or prospective study. Due to the small sample size, the findings may not be representative of the population, although they were sufficient to conduct validation analyses using *t*-tests. Moreover, the reliance on self-reported measures may introduce response biases, and the cross-sectional nature of the data precludes causal interpretations. Therefore, these findings should be viewed as a preliminary assessment of a phenomenon that warrants further investigation in a subsequent study utilizing a representative sample. Additionally, the questionnaire was developed and tested exclusively in Italy, which may limit the external validity of the findings. Cultural, social, and economic differences could affect the generalizability of the results to other populations. Therefore, these findings should be viewed as a preliminary assessment of a phenomenon that warrants further investigation in a subsequent study utilizing a representative sample and cross-cultural validation.

## 5 Conclusion

Reproductive attitudes not only significantly shape demographics, but also offer insight into the perspectives of the younger generation regarding the future. Hence, assessing how climate change affects reproductive attitudes becomes crucial.

The results of the present study, which link lower levels of reproductive attitudes to eco-anxiety, eco-paralysis, and depression, underscore the relevant societal impact of climate change in shaping the current and future demographic decline in Italy and the consequent need to consider these aspects in policymaking. Additionally, the mediation role of eco-paralysis between functional/cognitive impairment and climate change-related reproductive attitudes supports this assertion.

These insights have important implications for developing targeted interventions aimed at alleviating eco-paralysis and promoting sustainable behavior in the face of environmental threats. Future research should determine effective interventions to counter eco-paralysis and the psychological benefits of those interventions in reproductive attitudes and beyond the decision to have children.

To expand on this research, future studies should investigate the correlations between reproductive attitudes, eco-anxiety, and eco-paralysis, as well as explore the moderation model proposed by our study in the Italian context and in other countries with different cultural or economic realities. Moreover, given that our study population was mainly female, future studies should investigate male attitudes regarding reproductivity in relation to climate change more thoroughly.

## Data availability

The datasets generated during and analyzed during the current study are available from the corresponding author upon reasonable request.

## Funding

The authors declare that no funds, grants, or other support were received during the preparation of this manuscript.

## CRediT authorship contribution statement

**Matteo Innocenti:** Writing – review & editing, Writing – original draft, Methodology, Conceptualization. **Gabriele Santarelli:** Writing – original draft, Validation, Methodology, Investigation, Formal analysis, Data curation. **Chiara Comerci:** Writing – review & editing, Visualization, Formal analysis. **Niccolò Carluccio:** Writing – original draft. **Enrico Anzaghi:** Writing – original draft. **Chiara Cadeddu:** Writing – review & editing, Validation, Supervision, Project administration.

## Declaration of competing interest

The authors declare that they have no known competing financial interests or personal relationships that could have appeared to influence the work reported in this paper.

## Appendix Supplementary materials

Image, application 1
