# Supplementary material for: Development and validation of the climate change-related reproductive concerns scale (CCRCS)
Source: J Clim Chang Health. 2024 Oct 11;20:100351. doi: 10.1016/j.joclim.2024.100351 (PMC12851318; doi:10.1016/j.joclim.2024.100351)
Supplement: Supplementary file 1 [file mmc1.docx]

**APPENDIX A**

You will be given a series of 10 statements. Please indicate how much you agree with each of them. Choose the answer more appropriate for you: 1 = Strongly disagree; 2 = Disagree; 3 = Neither agree nor disagree; 4 = Agree; 5 = Strongly agree.

*Verranno presentate 10 affermazioni, la preghiamo di indicare il suo grado di accordo. Inserisca la risposta che ritiene più opportuna: 1 = Fortemente in disaccordo; 2 = In disaccordo; 3 = Né in accordo né in disaccordo; 4 = In accordo; 5 = Fortemente in accordo*

1. I don’t want to have children: the main reason is that they’ll grow up in a world ruined by climate change.

*Non voglio fare figli: il motivo principale è che cresceranno in un mondo rovinato dal cambiamento climatico.*

1. I'm not sure if I want to have children, but I know that climate change decreases my confidence in wanting to have them.

*Non so se voglio avere figli, ma so che il cambiamento climatico diminuisce la mia sicurezza nel volerli fare*

1. I am not afraid to have children because I don't think future generations will be significantly impacted by climate change

*Non ho timore a fare figli, perché non penso che le generazioni future saranno impattate in modo significativo dal cambiamento climatico*

1. Even though I fear the effects of climate change on future generations, I still want to have children in the future.

*Anche se ho timore degli effetti del cambiamento climatico sulle generazioni future, vorrei comunque avere dei figli in futuro*

1. The amount of emissions I would create by having children generates too much guilt for me to do so; therefore, either I won't have any, or I'll adopt one.

*La quantità di emissioni che creerei facendo dei figli mi genera troppi sensi di colpa per poterli fare; perciò, o non ne avrò, o ne adotterò uno*

1. One more or one less person doesn’t change the climate, so the choice to have children or not has nothing to do with climate change.

*Una persona in più o in meno non cambia il clima, quindi la scelta di avere o meno figli non ha a che fare con il cambiamento climatico*

1. Despite being aware that one more or one less person doesn't change the climate, I still choose not to have children.

*Nonostante sia consapevole che persona in più o in meno non cambi il clima, io scelgo comunque di non fare figli*

1. Climate change is not our direct responsibility but of those who produce emissions, so I will not give up having children to repair damage caused by others.

*Il cambiamento climatico non è responsabilità diretta nostra ma di chi produce emissioni, pertanto io non rinuncerò a fare figli per riparare a danni causati da altri*

1. Throughout my life, I often find myself asking the existential question of whether to have children or not because of climate change.

*Nel corso della mia vita mi ritrovo molto spesso a pormi la domanda esistenziale sul fare figli o meno, per via del cambiamento climatico*

1. I often discuss with my partner the difficulty of having children in a world impacted by climate change.

*Discuto spesso con il/la mio/a partner sulla difficoltà di fare figli in un mondo impattato dal cambiamento climatico*

During the preparation of this work the authors used the AI assistant “Erudite” in order to improve the readability of some parts of the manuscript. After using this tool, the authors reviewed and edited the content as needed and take full responsibility for the content of the publication. The AI tool was not used for the creation of the instrument.
